# Culture and pandemic control at cross-roads: navigating the burial guidelines for COVID-19-related deaths in a Ghanaian setting

**DOI:** 10.1186/s12913-023-09421-8

**Published:** 2023-05-23

**Authors:** Dorothy Takyiakwaa, Derek Anamaale Tuoyire, Susanna Aba Abraham, Elizabeth Ama Agyare, John Oti Amoah, Akosua Agyeiwaa Owusu-Sarpong, Kizito Omona, Dorcas Obiri-Yeboah, David Teye Doku

**Affiliations:** 1 Department of Sociology and Anthropology, University of Cape Coast, Cape Coast, Ghana; 2 Centre for Gender Research, Advocacy and Documentation, University of Cape Coast, Cape Coast, Ghana; 3 Department of Community Medicine, School of Medical Sciences, College of Health and Allied Sciences, University of Cape Coast, Cape Coast, Ghana; 4 Adult Health Department, School of Nursing and Midwifery, College of Health and Allied Sciences, University of Cape Coast, Cape Coast, Ghana; 5 Clinical Microbiology/Public Health Unit, Cape Coast Teaching Hospital, Cape Coast, Ghana; 6 Microbiology and Immunology Department, School of Medical Sciences, College of Health and Allied Sciences, University of Cape Coast, Cape Coast, Ghana; 7 Regional Health Directorate, Central Region, Cape Coast, Ghana; 8 Faculty of Health Sciences, Uganda Martyrs University, Kampala, Uganda; 9 Department of Population and Health, College of Humanities and Legal Studies, University of Cape Coast, Cape Coast, Ghana

**Keywords:** COVID-19 burial rites, Culture and pandemic control, Qualitative study, Ghana

## Abstract

**Background:**

Despite the large volume of scientific evidence on the rapid spread of the COVID-19 pandemic and associated high morbidity and mortality, little is known about the sociocultural disruptions which ensued. The current study explored the nuanced navigation of the COVID-19-related death and burial protocols and its impact on traditional burial and funeral rites in Ghana.

**Methods:**

This qualitative study was based on the ‘focused’ ethnographic design. Data were collected using key informant interviews from nineteen COVID-19-related bereaved family members and public health officials involved in enforcing adherence to COVID-19-related death and burial protocols in the Cape Coast Metropolis of Central region of Ghana. Recursive analysis was conducted to generate the themes and sub-themes from the data.

**Results:**

The overarching theme was “Uncultural” connotations ascribed to the COVID-19-related death and burial protocols. The COVID-19-related death and burial protocols were ubiquitously deemed by participants to be ‘uncultural’ as they inhibited deep-rooted indigenous and eschatological rites of separation between the living and the dead. This was fueled by limited awareness and knowledge about the COVID-19 burial protocols, resulting in fierce resistance by bereaved family members who demanded that public health officials release the bodies of their deceased relatives. Such resistance in the midst of resource limitation led to negotiated compromises of the COVID-19-related death and burial protocols between family members and public health officials.

**Conclusions:**

Insensitivity to socio-cultural practices compromised the implementation of the COVID-19 pandemic control interventions, particularly, the COVID-19-related death and burial protocols. Some compromises that were not sanctioned by the protocols were reached to allow health officials and families respectfully bury their dead. These findings call for the need to prioritize the incorporation of sociocultural practices in future pandemic prevention and management strategies.

## Introduction

The Coronavirus disease 2019 (COVID-19), which has since March 2020 been declared a pandemic by the World Health Organization (WHO), is considered as one of the greatest public health threats in modern history [1, 2]. The rapidity and geographical spread of COVID-19 resulted in unprecedented high morbidity and fatality, as well as disruption in socio-economic and socio-cultural endeavors [3, 4]. Globally, over 700 million people had been infected by March 2023, with over five million deaths [5]. With over nine million infections and at least 175,315 deaths, Africa is among the continents with the lowest COVID-19 related morbidity and mortality [5]. Within the African region, however, Ghana is ranked among the top twenty countries negatively impacted by the COVID-19 pandemic [6]. The number of confirmed COVID-19 cases in Ghana as of 21st March 2023 was estimated to be 171, 288, with resultant deaths totaling 1,462 [7].

In the Ghanaian culture, rites for the dead are considered to be of significant importance and are typically characterized by a series of rituals believed to be the only culturally acceptable way of elevating the dead to ancestral status, as well as ensure spiritual blessings for the bereaved family and entire community [8, 9]. Any compromises in the performance of these rites are believed to make the spirit of the dead vengeful towards the bereaved family or community [8, 10].

Similar to many countries around the world, Ghana’s public health response to the pandemic was informed by the scientific evidence on the mode of spread of the disease and the public health interventional guidelines established by WHO [2].

In Ghana, as part of the measures to curb the spread of the disease, the handling and burial of the bodies of those who succumbed to COVID-19 were to be taken over by the state. Trained environmental health officials (Transition Teams) were responsible for preparing and packaging such “contagious” bodies for burial. In addition, the government also introduced a new law known as the Restrictions Act. The Restriction Act, among other things, placed a ban on all forms of public mass gatherings including the observation of funeral rites which have very profound social and cultural significance among Ghanaians and Africans in general [8].

These restrictions struck the core of the social and cultural fabric of the Ghanaian society the hardest [8] as they meant that family members were unable to perform funeral and burial rites deemed essential for ensuring the safe transition of the deceased to the ancestral world [8]. As such, media reports suggest that the COVID-19 induced restrictions to death and burial rites were viewed across the Ghanaian society as a violation of the deep-rooted cultural prescriptions for the dead.

In particular, the alterations to traditional cultural rites pertaining to the handling of the dead, funeral and burial rites brought forth by the COVID-19 preventive measures were considered intolerable by many [8].

However, there is a dearth of empirical knowledge regarding how the said guidelines affected the socio-cultural practices relating to burial and funeral rites for relatives who died of COVID-19 related causes. This study sought to explore their experiences while navigating through the processes with public health officials responsible for the enforcement of the guidelines as well as the latter’s own experiences in enforcing the restrictions and guidelines.

## Methods

### Study design and setting

The study adopted a ‘focused’ ethnographic design, a pragmatic form of ethnography [11, 12], to explore the shared cultural practices and meanings of the families and health workers providing burial services to patients who died of COVID-19. The study was conducted in the Central Region of Ghana. Data were collected from health workers who were members of the COVID-19 taskforce of the Central Regional Health Directorate and, adult family members of persons whose deaths were officially certified as caused by COVID-19 infection.

### Sampling approach

The study employed a multistage sampling approach. It began with purposive sampling of the health facility and units because of their specific mandate to implement the burial protocol. Subsequently, we conveniently sampled the health workers who were members of the COVID-19 taskforce and were available in the facility at the point of data collection. Consequently, names and contact details of family members or next of kin of COVID-19-related deceased persons were obtained from the selected health facility in the Cape Coast Metropolis. Preference was given to the family members or emergency contacts who were most involved in the burial processes. The snowballing technique was utilized where some of the family members or next of kin who felt they could not provide detailed information on the issue referred us to other information-rich family relations. Thereafter, COVID-19 taskforce members from the Environmental Health Unit who participated in at least two of such burials were purposively sampled as key informants. A total of nineteen participants comprising thirteen family members or next of kin of COVID-19-related deceased persons and six COVID-19 burial taskforce members were recruited. Recruitment ceased at the point of data saturation [13].

### Data collection

Data collection took place between May and July 2022. Both written and verbal informed consent from participants were sought. Data were collected through key informant face-to-face in-depth interviews using semi-structured guides in English and Fante^1^ and tape-recorded. Reflections from the key informants were also recorded in field notebook. On the average, the interviews lasted for 30 min. The data was transcribed verbatim, translation of interviews conducted in Fante into English was done, and back translated to ensure that the meanings participants ascribed to their experiences were not lost during the translation process. The second source of data was the national and local protocols and documents that prescribe the process of burials for COVID-19 certified deaths. These methods were complimented by the researchers’ field notes and reflections of the field interviews [14, 15].

### Data processing and analysis

Recursive analysis was conducted guided by the steps proposed by Polkinghorne and Arnold [16]. To begin with, the printed transcripts were read while listening to the tapes concurrently to ensure familiarization with the data. Some of the researchers are native speakers and writers of the Fante language. Back-translation, researcher triangulation and intercoder agreement ensured that the semantic and meanings were not lost or misinterpreted. During this process, the key concepts in the interview questions were applied deductively to the transcripts, and statements of interest were highlighted. An abstractive approach was applied to categorize the data. This involved transferring of the highlighted statements into a table, each placed under the key concept it aligned with. Similar topics were combined to form the sub-themes. After reaching consensus among team members, the table became the codebook that was later applied to the other transcripts for data abstraction. The overarching theme was developed by comparing the sub-themes and identifying and condensing similarities, while returning to the original transcripts for validation of the meanings [16].

### Ethical considerations

Each family member or next of kin was contacted to explain the purpose of the study and to solicit their participation. Those who consented to participate were recruited and appointments were made for data collection.

## Results

### Demographic characteristics of respondents

A total of nineteen (19) respondents comprising 17 males and 2 females were interviewed. Ten of the participants worked in the formal sector, the remainder were a mix of those in the informal sector, self-employed, pensioners or unemployed. In terms of age, the range was between 34 and 75 years (see Table 1).

**Table 1:** Demographic characteristics of respondents

No.	Pseudonym	Sex	Age	Occupation
**Family Members**				
1	Afia	Female	N/A	Teacher
2	Mensah	Male	43	Lecturer
3	Bob	Male	N/A	Unemployed
4	Egya	Male	60	Accountant
5	Main	Male	75	Pensioner
6	Yaw	Male	N/A	Driver
7	Papa	Male	43	Surveyor
8	Kojo	Male	66	Pastor/Proprietor
9	Pee	Male	N/A	Pensioner
10	Kwabena	Male	63	Pensioner
11	Efua	Female	51	Model
12	Kweku	Male	58	Entrepreneur
13	Prempeh	Male	37	Unemployed
**Health Workers**				
14	May	Male	35	Clinician
15	Joe	Male	41	Mortuary staff
16	Oboy	Male	60	Mortuary staff
17	Kobina	Male	N/A	Mortuary staff
18	Yaw	Male	60	Environmental Officer
19	Williams	Male	N/A	Environmental Officer

### Emerging themes

The overarching theme which emerged from the analyses was the ‘Uncultural’ connotations ascribed to the COVID-19 burial protocols/guidelines”. Underlying this overarching theme were three sub-themes, namely: (1) Varied depths of knowledge of Ghana’s COVID-19 burial protocols (2) Perceived Cultural aberrations of the COVID-19 burial protocols/guidelines, (3) Attitudes towards Covid-19 burial protocol implementation. This is presented in Table 2.

**Table 2:** Emerging themes and sub-themes

Overarching theme	Subthemes		Sub-categories
‘Uncultural’ connotations ascribed to the COVID-19 burial protocols/guidelines	Sub-theme 1	Varied depths of knowledge of Ghana’s COVID-19 burial protocols	
	Sub-theme 2	Perceived cultural aberrations of the COVID-19 burial protocols/guidelines	*Perceived neglect of Culture in COVID-19 burial processes*
			*Implications of the ‘uncultural’ burial practices*
	Sub-theme 3	Attitudes towards COVID-19 burial protocol implementation	*Resistance amidst compromise of COVID-19 burial protocols*
			*A negotiated compromise of COVID-19 burial protocols*

### SUB-THEME 1: varied depths of knowledge of Ghana’s COVID-19 burial protocols

From the narratives, participants had some knowledge about various aspects of the burial protocols. For the health workers, most of them had undergone training prior to their assignment as members of the task force and thus were well informed about the protocols. However, the family members expressed a lack of awareness and understanding of the restrictions on burial of deceased persons diagnosed with COVID-19. They asserted that much of the sensitization and information readily available focused on everyday personal hygiene practices, and not details about the burial prescriptions such as the confiscation of bodies.

*“The way they [health workers] bury the dead when the person dies of COVID-19, they didn’t educate us on that. So, when it happens like that, what you will hear is that you will not be allowed to see your deceased when they want to bury him. All that you will hear is that, they have gone to bury him and even the place they went to bury him, you won’t even know. So, with that, they didn’t really educate us on that”***(Kweku, male, family member)**

It was obvious that, prior to the death of their own relatives, that the cultural implications of enforcing the COVID-19 burial protocols had not been discussed or explored amongst the family or the community at large. Their main source of knowledge had been information circulating in the community grapevine about how other people’s deceased relatives who had died as a result of COVID-19 had been buried. This lack of information impacted the families’ preparedness and participation in the burial processes and this resulted in the feeling of exclusion and the neglect of their culture.

*“Hmmm, for the COVID-19, it is the government who buries those bodies. You [families] don’t have the right to do whatever you want to do… Even when they are going to bury your deceased, they can call you or they can ignore calling you. So, if the family wants to do something [funeral], they will have to do it without the body”***(Yaw, Male, family member)**

### **SUB-THEME 2**: perceived cultural aberrations of COVID-19 burial protocols/guidelines

Despite the generally dynamic, complex and multifarious Ghanaian socio-cultural landscape, there was convergence from the narratives of respondents regarding importance and obligatory nature of rites associated with death, mourning and burial.

#### Perceived neglect of culture in COVID-19 burial processes

All the narratives indicated that the COVID-19 burial protocols ignored the culture and practices prevailing in the communities. The health workers also acknowledged that the burial protocols were at variance with the beliefs of the communities in which they worked and belonged.

*“The government employed some people to issue [a statement] that the burial of these bodies [persons who died from COVID-19] should be done in a particular way to prevent the spread of the disease. But… our traditions and culture don’t say or support the new way”***(Kobina, Male, mortuary staff)**

This perceived blatant neglect of culture underscored the families’ feelings of being disrespected and bred the sense of an imposition of foreign burial processes on them.

*“With COVID-19, what I saw wasn’t good. It’s not something that we should do. We have our culture and the Whites have their culture. In our culture... the way they buried our loved ones isn’t good. How they buried my brother was a disgrace and disrespectful, and it disgraced the town too”***(Papa Kweku, male, family member)**

All respondents acknowledged the need to perform the customary rites in respect and honor of the deceased who is believed to be capable of invoking punishment or blessings on the living family members, depending on the extent to which the prescribed socio-cultural rites for the dead are performed satisfactorily. As such, the COVID-19 guidelines which denied family members access to the body of their COVID-19 related deceased for the performance of the required customary death and burial rites were viewed as a huge socio-cultural affront. The restrictions meant that important rituals such as libation, washing and dressing the body, keeping wake and other specific rites at the grave site could not be performed. Various cultural connotations were ascribed to the non-performance of death and burial rites as described in the following excerpts.

*“…pouring of libation which is mainly done, first of all, to pray that the dead who are transiting to the other world should pave way for those who are still alive when their time is up and also to intercede for them so that the ancestors will protect them and make their works successful”***(Egya, male, family member)**

The sentiments expressed by respondents about what constitutes proper death and burial rites transcended beyond traditional Ghanaian socio-cultural ascriptions and practices to include religious or faith-based rites for the deceased. In other words, the death and burial restrictions imposed as a result of COVID-19 equally impeded adherence to religious prescriptions believed to cleanse the deceased and make them acceptable to the creator in heaven. One participant narrated how the important Islamic ritual washing for the dead could not be performed for his brother:

*“And before you could bury a Muslim, there is special bath done for the deceased…and the bath goes with some level of hope and belief… it is a special bath for Muslims…but my brother didn’t have that opportunity”***(Bob, male, Muslim, family member)**

A self-reported Christian also intimated that:

*“Well, I come from a Christian background… we [families] dress the deceased and put it in the coffin chosen by either the wife or the children, we lay the person in state overnight and in the morning, we take the person to the church for the burial service and then the minister of the church will bury the person and we are there to see the coffin is covered”***(Main, male, Christian, family member)**

#### Implications of the ‘uncultural’ burial practices

Participants reported several implications of ignoring the cultural requirements for the burial of their family members on both the living and the dead.

For some respondents, not being given the opportunity to perform the required rites for their relatives who died from COVID-19 related causes meant that their deceased was deemed a nonentity who could not be honored in death and whose fate could be likened to that of an aborted child.

*“In our culture, if they [the family] don’t perform any service or [traditional] rites for you when you die, then you’re the same as an aborted child. That is to say that you were of no value”***(Efua, female, family member)**

### SUB-THEME 3: attitudes towards COVID-19 burial practices implementation

There was resistance from family members towards the implementation of the COVID-19 burial practices. Over time, engagements resulted in compromises to accommodate both the public health concerns and cultural demands on handling the dead and burials, even though some families accepting the implementation of the protocols from the outset.

#### Resistances amidst compromise of COVID-19 burial protocols

Some family members resisted COVID-19 related burial protocols which were considered as an aberration of the customary or religious rites required to pave way for the smooth transition of the dead to the spiritual world. This state of affairs even led to instances of violence and push-backs towards the health workers and transition teams as averred in the following excerpts.

*“…the majority of them [families] were not in agreement with the laid down procedures; that the body cannot be released to them, they cannot bury at the site they prefer and the date they prefer so usually that was the major difficulty or challenge”***(May, male, pathologist)**

*"After discussions with the family and giving them the necessary rules, you [health worker] get to the point and the family wants to [physically] attack you, thinking that it is the Environmental Health Officers who diagnosed the deceased as COVID-19 patient"* **(William, male, environmental health worker)**

*“The family members resist and fail to accept that the relative died of COVID-19. So, they won’t allow such burial [COVID-19 Burial protocol] of their relatives. This led to a conflict between the hospital staff and the family. As a result of that, those who are quick tempered start to curse us”* **(Kobina, male, mortuary staff)**

Some participants expressed their lack of confidence in the test results and claimed that some health workers had personal interests, and that became the basis for their vehement resistance of the COVID-19 burial protocols.

*“So, they [family members] attacked, saying that the person did not die from COVID-19 but because we have an interest that’s why we declared the deceased a COVID-19 victim”***(Yaw, male, environmental health worker)**

*“…I was told that the World Bank donated some money which could only be used on condition that you [health workers] testify that A, B and C tested positive… So, they wanted to use the money, so, if you’ve contracted COVID-19 or not, they record that the person died of covid”* **(Mensah, male, family member)**

There were expressions of doubts about the efficacy of the testing mechanism, and indeed the entire health system. Family members of the deceased therefore argued that the test samples and results are sometimes wrongly assigned.

*“Following the death of my brother, the doctor directed us to the Environmental Health Officers to discuss the procedure for burial. When we went, we got to know that they [Environmental Health Officers] had called the deceased’s next of kin that had a similar name to mine but the phone number they used and the surname were not mine. So, my relatives and I were confused. They interchanged someone with us. We argued our case but there was no change. So that was what happened the very day”***(Pape Kweku, male, family member)**

Other interlocutors intimated that the actions of some of the health workers and taskforce implementing the COVID-19 burial protocols fueled their doubts about whether the deceased actually died from COVID-19. Participants narrated how some transition team members handled the “so-called” COVID-19 bodies without wearing the required PPEs. To them this was a source of suspicion about whether indeed their relative had died of the disease, and therefore did not see why their relative’s dead body should not be released to them for the befitting customary death and burial rites to be performed.

*“They [mortuary workers] were not in any face masks… If they say that the deceased died of COVID-19, and they say it is an infectious disease, and the dead person is still contagious, how come they don’t wear face masks and they are not infected”? ***(Bob, male, family member)**

*“When we went for the death certificate, that’s when we found out he died of COVID-19. But we didn’t get any report that the COVID-19 test they did was positive. That’s why I was challenging them”* **(Mensah, male, family member)**

#### A negotiated compromise of COVID-19 burial protocols

Although the COVID-19 burial protocol required that designated public health officials (transition teams) have the sole responsibility for preparing, and burying the bodies of those who died of COVID-19 related cause, the narratives from study participants reveal otherwise. Instances of discretion were reported and observed mainly in relation to preparation of the deceased and the burial process to accommodate the cultural demands of the families.

*“I’m a Muslim, so we gave them a white cloth to wrap my brother. One of my uncles also gave them instructions during the bath. They [morticians] accepted our demands and wrapped him with the cloth as we wanted and put him in the body bag. We pleaded with them to give us some five minutes, relatives, friends and sympathizers were all there [at the morgue and we prayed. After that we followed them [COVID-19 team] with our cars to the burial site…. At the cemetery, we stood at a distance but showed them that the head should be at their left and other rites according to how Muslims bury their dead. So, we prayed for him and then returned home” ***(Bob, male, family member)**

There seemed to be a sort of negotiated trade-off between families of the COVID-19 dead and the public health officials. For instance, some families were granted requests to bury their dead relatives in cemeteries of their choice, if they were able to provide the needed burial logistics and PPEs which the public health official lacked.

*“If you [family] want to be involved, then you have to provide the necessary things for the transportation. You need a police escort, fuel the vehicles and you also need to make sure that when we get to the place, nobody handles the body. It should be done like how it is stated in the protocol … and we don’t want too many people around; only selected family members. And then, we made them sign a document”***(William, male, environmental health worker)**

One motivation that engendered a compromise of the COVID-19 burial protocols was the seemingly reduced perception of severity and risk associated with COVID-19 over time. This was succinctly expressed by a public health official who considered the threat of COVID-19 to be relatively low compared with Ebola:

*“The COVID-19 burial protocol was strictly according to Ebola protocols. However, when new discoveries were made, it was somehow relaxed so that families’ requests for family burial were accepted. We sort of compromised with them under strict supervision because we realized that it [COVID-19] isn’t as fearful as Ebola. So, we put some protocols in place for the transfer of the body to that district”***(William, male, environmental health worker)**

While the involvement of family members in the process afforded many to pay their last respects in grieve and have some closure, instances of disregard for the COVID-19 burial protocols were also unearthed; such as family members burying their deceased without the presence of the task force and also at their preferred burial sites.

*“So, I had to do whatever was necessary to just give my sister a befitting burial. In short, the body was released to us. Yeah. We did that…we went through the state processes of getting permission to bury my sister - pay the prescribed fees…we satisfied all the conditions and they gave us a space…so in the end the environmental officers were not there”***(Kweku Mensah, male, family member)**

Some narratives inferred claims of unapproved fees and extortion from relatives of those who died from COVID-19 before releasing such bodies which would otherwise have remained in the domain of public health officials in accordance with the protocol.

*“The thing [COVID-19] is there but at some point, it was used for personal gains. They took money from me and then released the body to us. It’s not good”* (**Kweku, male, family member)**

The results also suggest interference in the implementation of the burial protocols from “highly placed” persons in society to circumvent the COVID-19 burial protocols.

*“For some they even came with letters asking for the body to be released. I remember there was one case that the letter came from very high above so the body was just released the same day to the family” ***(May, male, pathologist)**

However, for those who did not have access to the persons in authority, there was reluctant acquiescence which resulted in their deceased relatives’ burial being guided by the COVID-19 protocols. Nonetheless, they emphatically registered their displeasure about not being allowed to perform the necessary customary burial rites. Some shared:

*“They said my elder sister died of this disease [COVID-19] … So, I said, master I won’t believe whatever you are saying because I know that my sister has a problem, hypertension. So, he [health worker] said, we beg you just listen to whatever we are saying. Because if you try to stretch the issue, they will use their power because of the President’s directives… So, if we want to behave this way, they will also make it very rigid for us which will cost us not having access to the body of my sister and will bury her strictly in accordance with the directive”***(Kweku Mensah, male family member).**

Other participants also conceded to the implementation of the burial protocols by ceding power to the experts.

*“They [health workers] are the experts and they are telling you that this is the condition. I only received a report when they gave me the death certificate and they indicated that it was a severe COVID-19”***(Yaw, male, family member)**

## Discussion

The current study applied a qualitative approach in exploring the perspectives and experiences of those who lost relative(s) to COVID-19 related cause and the public health officials responsible for ensuring compliance with the COVID-19-related death and burial protocols. This study revealed that the COVID-19-related death and burial protocols were ‘uncultural’ as they violated customary traditional culture. The study also found limited awareness and knowledge about the COVID-19 burial protocols which resulted in fierce resistance by bereaved family members. This led to negotiated compromises of the COVID-19-related death burial protocols between family members and public health offices which consequently violated the public health interventions aimed at the control and management of the pandemic.

Culture is a mosaic of shared meanings, rituals and their symbolisms, and expectations. Societies usually have agreed upon ways and expectations of how certain important aspects of social life are planned, organized and executed, including burial rites and funerals. Similar to other studies [8, 17–19] across Africa, our results revealed that the COVID-19-related death and burial protocols were deemed to be ‘uncultural’ as they violated customary traditional culture and even religious rituals for the dead. However, missing in these earlier studies are the cultural contextual nuances in the nature of resistances to the COVID-19 burial protocols. Our study highlighted context-specific resistances such as rejection and manipulation of test results, physical violent attacks on health workers and invocation of curses on the health workers. This impacted the implementation of the burial protocols and thus posed a challenge for reducing COVID-19 infections. Again, the novelty of this study is the fact that previous studies [20] mainly reported the absence of cultural sensitivity and considerations related to COVID-19 burial protocols. We have presented in this paper how this absence impacted the implementation of the COVID-19 burial protocols. African and for that matter the Ghanaian funerals are typically grounded in traditional and religious practices and ceremonial grandeur as exemplified in research on the Akan burial conditions and rites [19]. The COVID-19 burial protocols meant that the multiple levels (e.g., pre-burial, burial, post-burial) and rites (e.g., libation, preparing bodies, widowhood rites, lying-in-state, file past, etc.) could not be performed for those who died from the pandemic. Many understood the guidelines as cultural aberrations which had dire implications for the successful passage of their dead to the ancestral world, and subsequent ramifications on the living [19, 21, 22].

Although the WHO recommends that countries adapt the COVID-19 death and burial guidelines to ensure they are culturally sensitive, it seems this call was not heeded in all settings.

It emerged from the narratives that most family members had very limited knowledge about the COVID-19 burial guidelines compared with the general PHSM relating to hygiene, social distancing and wearing of face masks. Indeed, the COVID-19 sensitization and educational campaign efforts in Ghana and elsewhere therefore seem to have overemphasized more on the personal protective components of the PHSM with little attention given to other issues which borders on the socio-cultural values of the people such as burial protocols and the responsibilities or otherwise of family members of dead COVID-19 victims.

The study also revealed that in the space of limited information and awareness, misinformation, misunderstanding, mistrust, skepticism and even exploitation festered. Public health officials took upon themselves to fill the information gap about burial protocols by explaining the protocols to family members who were adamant under the circumstances of grief and despair. Prior public health education on aspects of the protocol especially those which bordered socio-cultural fabrics of society was missing. The negative perceptions and meanings drawn from the unprecedented COVID-19 burial restrictions and limited prior knowledge necessitated the resistance from family members who demanded the release of the bodies of their relatives who died of COVID-19 related causes for culturally dignified burials. Indeed, various conspiracy theories [23] including one that suggests that the pandemic is an avenue for the government to ‘make money’ from international bodies such as the IMF and the World Bank and their partners were commonly cited as underlying reasons for the fierce resistance to the burial guidelines.

As public health professionals struggled to balance the precautionary burial protocols with empathy for the dead as well as the grieving family members, compromises were negotiated. The findings suggest that families were allowed a level of involvement in the burial processes for their relatives who died from COVID-19 contrary to the guidelines, once they were willing to pay for some of the expenses and logistics for burial. This to some extent afforded family members the opportunity to ensure a more culturally dignified burial for the deceased and as a way of finding closure. Such compromises or modifications to burial protocols have been noted in the literature with respect to the 2014 Ebola epidemic [24]. Insufficient logistics for the COVID-19 related burial enforcement team as well as poor working conditions might have also contributed to this compromise.

The results also point to the exercise of discretionary power on the part of health officials as ‘street-level bureaucrats’ responding to each unique situation that confronted them instead of fully implementing the COVID-19 burial protocol. Tummers and Bekkers [25] assert that such discretion exercised by street-level bureaucrats can be positive or negative. According to Akosa and Asare [26], discretion exercised negatively can lead to discrimination, show of favoritism or disregard for the guideline. These were evident in our study as some health officials circumvented the protocols by releasing COVID-19 dead bodies to those ‘highly placed’ in society or those who were willing to make some payments. This resulted in inequalities in the treatment of bodies of those who died of COVID-19 related death on the basis of socioeconomic status of the victim or their family, as has been previously reported in similar epidemic or disaster situations [20, 27].

The strengths of this study were: (1) in the originality of the ethnographic methodological approach, which allowed the in-depth exploration of the cultural and contextual issues that emanated from the implementation of the COVID-19 burial protocols; (2) empirically, it also highlights novel findings on the nuances of resistance in the cultural context where the study was conducted. The limitations could be the researcher’s recall biases and social desirability biases, which were managed by researcher and data triangulation. Being a qualitative study, it findings may not be generalizable to other populations and contexts, but however insights from this study is important for similar research and context.

## Conclusion

The study demonstrates that the precautionary COVID-19-related death burial protocols which distanced family members from their relatives who succumbed to the disease were deemed culturally repressive as they inhibited deep-rooted indigenous and eschatological rites of separation between the living and the dead. The values and sociocultural significances placed on sacred obligatory death rites engendered tensions between COVID-19 related bereaved and public health professionals. In a bid to navigate through the tensions amidst resource limitations, burial teams negotiated compromises with family members in their struggle to maintain a balance between safety protocols, respectful rites for the dead and empathy for the bereaved. These findings reinforce calls for future pandemic strategies to first draw on expectations and sensitive cultural values of all stakeholders in all preventive strategies. It also highlights the need for adequate communication and dissemination of strategies in order to limit the space for discretionary implementation which is likely to lead to inconsistencies and misapplication of public health strategies. Over all, these findings have important implications for the preparedness for prevention and management of future pandemics.

## Data Availability

All the data have been used and presented in this manuscript. Any additional information needed can be obtained from the corresponding author upon a reasonable request.

## Footnotes

1 Predominantly spoken Ghanaian language in the study setting.

## List of abbreviations

COVID-19: Coronavirus disease 2019
DRIC: Directorate of Research, Innovation and Consultancy
IMF: International Monetary Fund
PHSM: Public Health and Social Measures
PPE: Personal Protective Equipment
WHO: World Health Organization
